# Testing Bayesian and heuristic predictions of mass judgments of colliding objects

**DOI:** 10.3389/fpsyg.2014.00938

**Published:** 2014-08-26

**Authors:** Adam N. Sanborn

**Affiliations:** Department of Psychology, University of WarwickCoventry, UK

**Keywords:** intuitive physics, Bayesian models, heuristics

## Abstract

Mass judgments of colliding objects have been used to explore people's understanding of the physical world because they are ecologically relevant, yet people display biases that are most easily explained by a small set of heuristics. Recent work has challenged the heuristic explanation, by producing the same biases from a model that copes with perceptual uncertainty by using Bayesian inference with a prior based on the correct combination rules from Newtonian mechanics (*noisy Newton*). Here I test the predictions of the leading heuristic model (Gilden and Proffitt, 1989) against the noisy Newton model using a novel manipulation of the standard mass judgment task: making one of the objects invisible post-collision. The noisy Newton model uses the remaining information to predict above-chance performance, while the leading heuristic model predicts chance performance when one or the other final velocity is occluded. An experiment using two different types of occlusion showed better-than-chance performance and response patterns that followed the predictions of the noisy Newton model. The results demonstrate that people can make sensible physical judgments even when information critical for the judgment is missing, and that a Bayesian model can serve as a guide in these situations. Possible algorithmic-level accounts of this task that more closely correspond to the noisy Newton model are explored.

## Introduction

The correspondence between people's intuitive physical judgments and Newtonian mechanics has long provided a fascinating window into how people understand the physical world. Despite people's demonstrated competence in dealing with a complex physical environment, there are mismatches between intuitive physics and Newtonian mechanics (Michotte, 1963; McCloskey et al., 1980; Todd and Warren, 1982). These mismatches have led to the conclusion that people use heuristics to guide behavior, rather than using the decision rules derived from an accurate conception of physics (Todd and Warren, 1982; Gilden and Proffitt, 1989).

An interesting case is mass judgments of colliding objects. In the usual mass judgment experiment, two objects start moving, make contact, and then move apart. The task of the participant is to judge which object was heavier, a task that is ecologically relevant and therefore one that participants should be able to perform (Gibson, 1966; Runeson, 1983). Despite this, participants show strong biases away from the predictions of Newtonian mechanics, most importantly the *motor object bias*, a bias to believe that the object initially moving faster is heavier (Todd and Warren, 1982; Gilden and Proffitt, 1989; Runeson et al., 2000). This bias has been explained by simple heuristics that incorrectly combine the available information. The leading heuristic model for this task was introduced by Gilden and Proffitt (1989) and is referred to here as G&P. This model combines two heuristics: looking for the object moving faster after contact and looking for the object that ricochets, both imperfect cues that an object is lighter.

However, the interpretation of the motor object bias has been challenged recently by a framework that combines Bayesian inference with prior beliefs based on the correct combination rules from Newtonian mechanics (*noisy Newton*; Sanborn et al., 2013). This approach also predicts a motor object bias, but does so as a result of plausible prior beliefs about velocities. Because the key behavioral evidence can be produced by two approaches with fundamentally different views of how the mass judgment task is done, this resurrects the original question: How well does people's behavior correspond to the rules of the physical world?

A key difference between the noisy Newton and G&P models is the level of analysis at which each is cast. The noisy Newton model gives a computational-level account of the data, describing what should be done to solve the task, while the G&P model gives an algorithmic-level account, describing what processes people use to solve the task (Marr, 1982). While just about any behavior can be produced at either level of analysis (algorithmic-level approaches that can closely mimic the noisy Newton are discussed below), the existing versions of the noisy Newton and G&P model make different predictions for when the mass judgment task is changed. A strong version of the noisy Newton model predicts that people will always match task demands, while a strong version of the G&P model predicts that people will be limited to using the heuristics that have already been hypothesized.

I chose to adjust the mass judgment task by making one of the objects invisible after they make contact, because as I show below, the two models make very different parameter-free predictions when one or the other final velocity is occluded. The final speed heuristic relies on a comparison of the two final velocities to one another, and the loss of final velocity information can mean the G&P model predicts chance performance. In contrast, the noisy Newton model combines the remaining information appropriately to produce above-chance performance, but predicts that the pattern of results will strongly depend on which final velocity is occluded. Below, I describe the task and the normative rule from Newtonian mechanics and the predictions the G&P model and noisy Newton model make. An experiment is presented that tests these predictions using different ways of removing final velocity information and it confirms the predictions of the noisy Newton model over the G&P model. These results show that people display good performance: they conform to task demands when information is missing and perform better than the G&P model predicts. The implication of the results is that algorithmic-level approaches need to more closely approximate the noisy Newton model in order to successfully predict human behavior, and algorithmic-level approaches that can successfully do so are discussed.

## Newtonian mechanics for colliding objects

**Figure 1:**
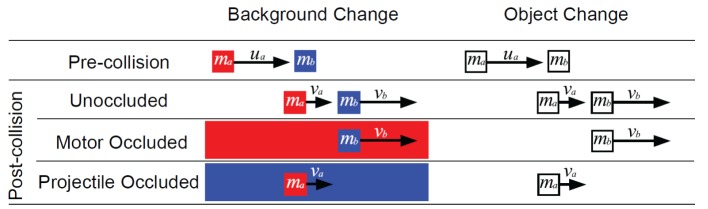
**Schematic of the trials shown in the experiment**. The pre-collision event occurred in every trial with only the mass ratio and *u*_*a*_ varying. Velocities to the right are defined as positive and to the left as negative. At contact, either the motor (motor occluded), projectile (projectile occluded) or neither object (unoccluded) disappeared. The method of disappearance differed in the background change and object change conditions.

Inspired by Michotte (1963), researchers have run a host of experiments investigating judgments of colliding objects. In its most common form, as shown in Figure 1, one of the objects, the motor object, *a*, begins moving with an initial velocity *u*_a_. The motor object comes into contact with an initially stationary object, the projectile object, *b*. After contact, the motor and projectile objects move apart with final velocities *v*_a_ and *v*_b_ respectively. Newtonian mechanics, assuming point masses and no external forces, gives the final velocities

(1) $$v_{a}\;=\frac{u_{a}\left(\frac{m_{a}}{m_{b}}-e\right)}{\frac{m_{a}}{m_{b}}+1}$$

$$v_{b}=\frac{u_{a}\frac{m_{a}}{m_{b}}\left(1+e\right)}{\frac{m_{a}}{m_{b}}+1}$$

where *m*_*a*_ and *m*_*b*_ are the masses of the motor and projectile objects respectively and *e* is the coefficient of restitution. The coefficient of restitution reflects the kinetic energy retained relative to the joint center of mass, ranging from *e* = 1, similar to colliding billiard balls, to *e* = 0, similar to colliding balls of wet clay.

The object with the greater mass can be determined from conservation of momentum using the initial and final velocities, and for all collisions in the experiment below

(2) $$m_{a}>m_{b}\;\text{if}\;\text{and only if }v_{a}+v_{b}>u_{a}.$$

The comparison of the sums of initial and final velocities provides a normative answer to the question of which object is heavier. However, the normative rule does not predict a motor object bias and it does not naturally give an answer if one of the final velocities is missing.

## Heuristic model predictions

The G&P model uses a combination of two heuristics to explain the motor object bias: the object that moves faster after collision is lighter and the object that ricochets is lighter (Gilden and Proffitt, 1989). The final speed heuristic produces a strong bias to pick the motor object and the ricochet heuristic allows the bias to be overcome when the projectile object is much heavier than the motor object. Further details are given in the Appendix in Supplementary material.

**Figure 2:**
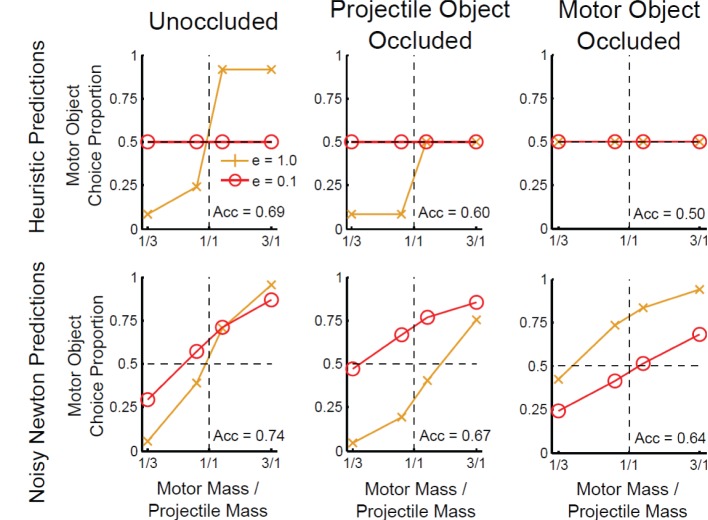
**Predictions of the heuristic and noisy Newton models for different types of occlusion conditions in Experiment 1**. The horizontal axis is the mass ratio of the motor object to the projectile object. The vertical axis is the proportion of trials on which participants decided the motor object was heavier. Each plot shows lines for two separate coefficients of restitution: *e* = 1 and *e* = 0.1. A bias is shown if the data lines do not cross the horizontal dotted line at the vertical dotted line. Acc refers to the predicted accuracy in mass judgment.

The G&P model relies on the availability of the velocities, mainly the final velocities, to produce an answer. In previous work, if not enough information was available to determine the ricochet heuristic, then that heuristic was not used (Gilden and Proffitt, 1994). As a result, I assumed that if either final velocity is missing, then the final-speed heuristic cannot be used^1^. If the final velocity of the motor object is missing, then there is no possibility of observing a ricochet either, and as no heuristics apply chance performance is predicted for all mass ratios and values of *e* (see Figure 2).

## Noisy newton model predictions

The noisy Newton model treats object masses as hidden variables that are inferred from noisy observations of the velocities. Combining the observed velocities with prior beliefs, Bayesian inference is used to calculate the probability that *m*_*a*_ > *m*_*b*_. The parameters used in Sanborn et al. (2013) that define the plausible prior distributions and psychophysically motivated noise were carried over to this work; details are given in the Appendix in Supplementary material. As in that paper noisy perception was approximated by noisy decision making—instead of feeding noisy values into the model and making a deterministic response, the noiseless values were fed into the model and the probability of the outcome was taken as the probability of response.

The motor-object bias is produced by plausible prior distributions on the velocities interacting with Newtonian mechanics. The prior distribution on *u*_*a*_ was set so that slower objects were considered more likely than faster objects, as motivated by velocity judgment experiments (Weiss et al., 2002; Stocker and Simoncelli, 2006). As the prior distribution on *u*_*a*_ is both narrower than the prior distribution on *v*_*a*_ + *v*_*b*_ and centered around zero, this results in a bias to believe *u*_*a*_ is less than *v*_*a*_ + *v*_*b*_. As can be seen in Equation 2, this bias in beliefs about the velocities is equivalent to a bias in believing the motor-object is heavier than the projectile object (Sanborn et al., 2013).

Figure 2 shows the predictions of the noisy Newton model for the occlusion conditions. Unlike the strong version of the G&P model, this model flexibly uses the available information to make inferences about the mass ratio, which allows the noisy Newton model to predict above chance performance if either final velocity is missing.

In addition, Figure 2 shows the how the predictions of the noisy Newton model depends on which final velocity is missing. First, depending on which final velocity is missing, there will be a reversal in whether *e* = 1 or *e* = 0.1 result in more choices of the motor object. This prediction follows from Newtonian mechanics: a larger *v*_*a*_ or *v*_*b*_ means a higher probability the motor object is heavier. Equation 1 shows that increasing *e* increases *v*_*b*_, but decreases *v*_*a*_. Thus, when the motor object is occluded a higher *e* results in a *v*_*b*_ which is more likely to exceed *u*_*a*_, and so by Equation 2 the motor object is predicted to be chosen more with *e* = 1. Conversely, in the projectile object occluded condition a higher *e* results in a *v*_*a*_ that is less likely to exceed *u*_*a*_, and thus the prediction that the motor object is chosen more with *e* = 0.1. I ran a parameter search across all positive values of the noisy Newton parameters for parameters that reversed the order of the effects, but none were found.

Second, in addition to the reversal in order, the noisy Newton model predicts that which object is occluded will determine whether the *e* = 1 condition produces a motor object or a projectile object bias. A motor object bias is predicted if the motor object is occluded, but a projectile object bias is predicted if the projectile object is occluded. This prediction is parameter dependent ^2^.

## Experiment

To test the predictions of the G&P and the noisy Newton model, the motor or the projectile object needs to be occluded after contact. This occlusion was performed as shown in Figure 1: either the occluder suddenly appeared at contact (background change) or one of the objects suddenly disappeared at contact (object change). Both types of occlusion were run in a between-participants design in order to make sure that the results did not depend on the details of the display.

### Methods

#### Participants

Forty-two participants were recruited from the University of Warwick community for this study. Each participant received £5 for one-half hour of participation. Participants viewed one of two types of occlusion: twenty participants viewed object change occlusions and twenty-two participants viewed background change occlusions.

#### Stimuli

Squares in the object change condition were white or gray and squares in the background change condition were red or blue. In both conditions squares were 1 cm in length. Movies started with the projectile object in the center. The motor object moved toward the projectile object until the edges of the two squares touched, then the objects immediately separated.

On each trial either the motor object, the projectile object, or neither object was occluded. In the object change condition, an occluded object was set to the black background color at contact, rendering it invisible. In the background change condition, the red and blue objects traveled along a white field until contact, at which time the field could change color to match one of the objects, rendering that object invisible.

The mass ratio of the heavier to lighter object was set to be either 1.25 or 3.0. On each trial, the heavier object was set to be the right or left object with equal probability. The initial velocity of the motor object (always the left square) was drawn on each trial from a uniform distribution that ranged from 1.91 to 4.45 cm/s in steps of 0.13 cm/s, while the initial velocity of the projectile object was always zero. The coefficients of restitution used were 1.0 and 0.1 and this parameter was a hidden variable: like the masses it was only discernable through the presented velocities. The final velocities of the two objects were calculated from Equation 1.

#### Procedure

Participants were instructed that the squares were blocks sliding along an invisible smooth surface, to keep their eyes on the left hand object, and to press a key corresponding to whichever block they thought was heavier. A trial would end automatically after one second, but participants could end the trial at any point by responding. No feedback was given to participants during the experiment.

A total of 192 trials were presented to each participant. There were twelve possible combinations of mass ratio, coefficient of restitution, and occlusion. One example of each combination was shown at the beginning of the experiment to familiarize participants with the displays. No feedback was given and these practice trials were discarded. The test trials consisted of 15 replications of each combination of mass ratio, coefficient of restitution, and occlusion with order of presentation randomized for each participant.

### Results

**Figure 3:**
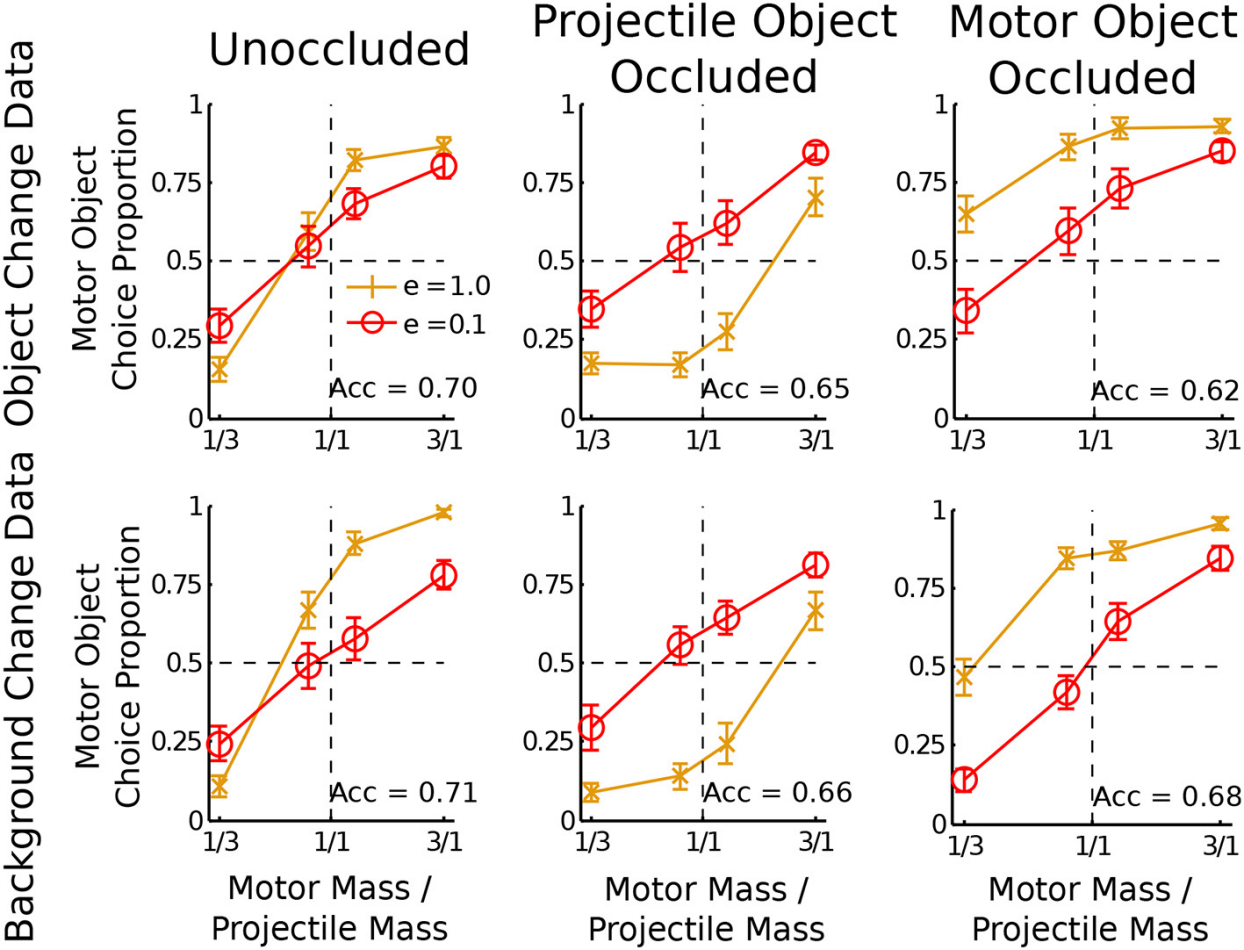
**Data from the object change and background change participants for different types of occlusion conditions**. The horizontal axis is the mass ratio of the motor object to the projectile object. The vertical axis is the proportion of trials on which participants decided the motor object was heavier. Each plot shows lines for two separate coefficients of restitution: *e* = 1 and *e* = 0.1. A bias is shown if the data lines do not cross the horizontal dotted line at the vertical dotted line. Acc refers to the accuracy in mass judgment.

Results are shown in Figure 3. Accuracy was examined with *t*-tests and Bayesian *t*-tests (Rouder et al., 2009; measured by the Bayes Factor (BF) likelihood ratio of the alternative model over the null model assuming width *r* = 1). For both the object change and background change occlusions, participants were significantly more accurate than chance when the motor object was occluded (*M*s = 0.62 and 0.68 respectively; *t*s = 5.7 and 12.1 respectively; both *p*s < 0.001; both BFs > 10^3^), supporting the noisy Newton model over the heuristic model. Participants were also significantly more accurate than chance when the projectile object was occluded (*M*s = 0.65 and 0.66 respectively; *t*s = 7.6 and 12.5 respectively; both *p*s <.001; both BFs > 10^3^).

The dependence of response bias on the final velocity occluded was examined by subtracting the proportion of motor object responses when *e* = 0.1 from the proportion of motor object choices when *e* = 1. For the projectile object occluded condition, both the object change and background change data showed more choices of the motor object for *e* = 0.1 than *e* = 1 (*M*s = −0.25 and −0.29 respectively; *t*s = −8.82 and −8.15 respectively; both *p*s < 0.001; both BFs > 10^5^). For the motor object occluded condition the opposite result was found: both the object change and background change data showed more choices of the motor object for *e* = 1 than *e* = 0.1 (*M*s = 0.21 and 0.27 respectively; *t*s = 6.65 and 11.2 respectively; both *p*s < 0.001; both BFs > 10^4^). This reversal confirmed the first specific prediction of the noisy Newton model.

A motor object bias was found in the motor object occluded condition: when $\frac{m_{a}}{m_{b}}=\frac{4}{5}$ and *e* = 1, participants chose the motor object more than chance for both the object change and background change data (*M*s = 0.86 and 0.84 respectively; *ts = 8.81* and 9.90 respectively; both *p*s < 0.001; both BFs > 10^5^). A projectile object bias was found in the projectile object occluded condition: when $\frac{m_{a}}{m_{b}}=\frac{5}{4}$ and *e* = 1, participants chose the motor object less than chance for both the object change and background change data (*M*s = 0.24 and 0.27 respectively; *ts = −3.88* and −4.03 respectively; both *p*s < 0.001; both BFs > 39). These biases confirmed the second specific prediction of the noisy Newton model.

A surprising effect occurred in the neither occluded condition. Previous experiments show a stronger motor object bias for lower values of *e* (Todd and Warren, 1982; Sanborn et al., 2013), an effect which was missing or perhaps reversed here. This was predicted by neither model and is a topic for future research.

The quantitative match of the model predictions to the data was assessed by finding the probability of the data in Experiment 1 with the parameters used to fit the data in Sanborn et al. (2013). For the object change data, the noisy Newton model predicted more accurately (negative log likelihood (NLL) of 937 where lower is better) than the G&P model (NLL = 1186). The noisy Newton model (NLL = 869) also predicted the background change data better than the G&P model (NLL = 1208).

## Discussion

The noisy Newton and G&P models are fundamentally different explanations of how people combine information to make mass judgments, but both have been able to explain the main features of existing data. To test them I introduced the novel manipulation of occluding one of the final velocities, a manipulation for which these models make strongly divergent predictions. The noisy Newton model predicted the experiment's results better than the G&P model.

**Figure 4:**
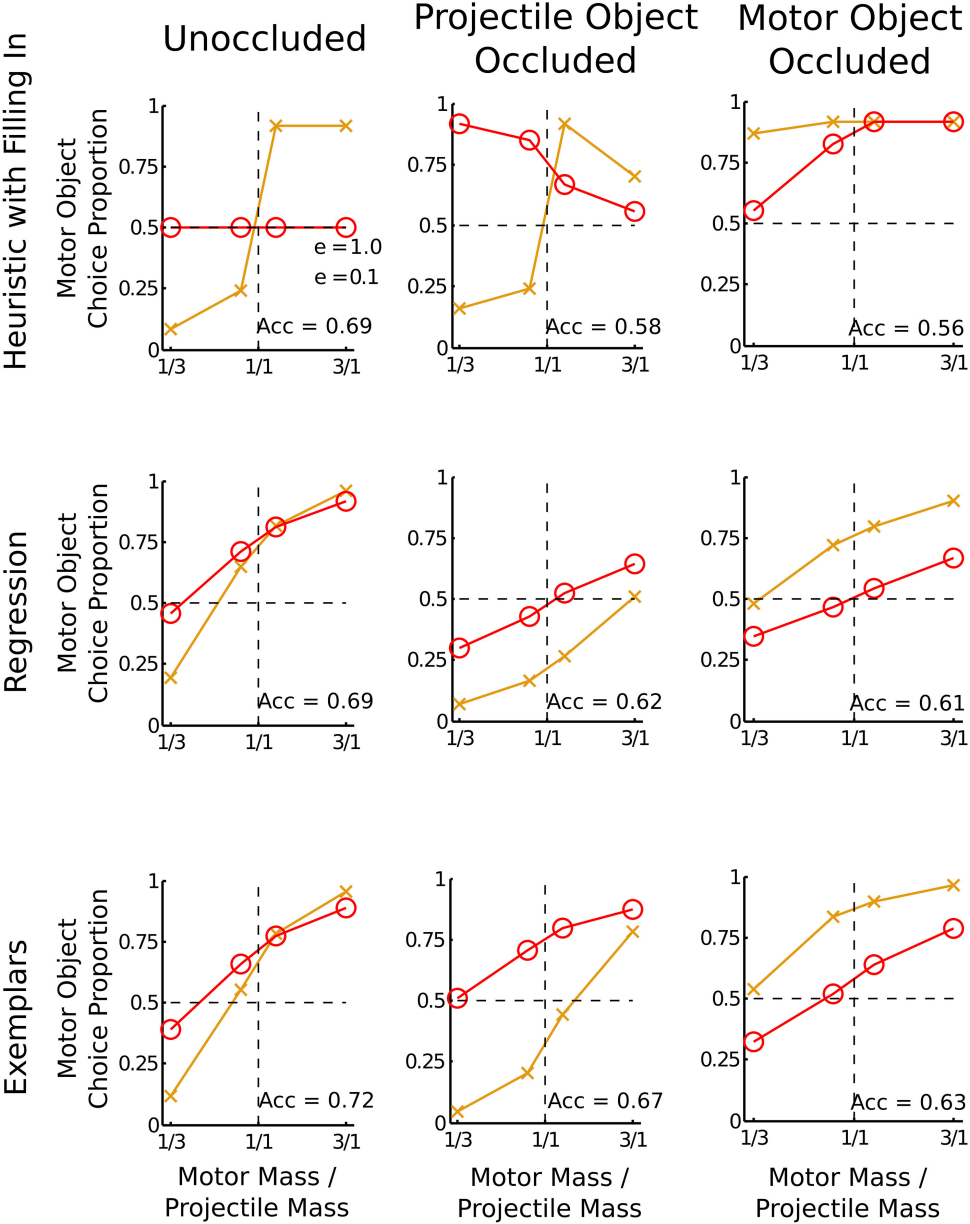
**Three alternative models for the occlusion conditions**. The horizontal axis is the mass ratio of the motor object to the projectile object. The vertical axis is the proportion of trials on which participants decided the motor object was heavier. Each plot shows lines for two separate coefficients of restitution: *e* = 1 and *e* = 0.1. A bias is shown if the data lines do not cross the horizontal dotted line at the vertical dotted line. Acc refers to the predicted accuracy in mass judgment. Each alternative model is described in the text.

There are many other algorithmic-level possibilities for how people make mass judgments, some that are closer to the G&P model and some that are closer to the noisy Newton model. One possibility is that a slight change to the G&P model would allow it to predict mass judgments with occluded final velocities. For example, instead of occlusion rendering heuristics inoperable, it is possible that participants fill in missing final velocity information with a plausible estimate. Assuming that the filled-in missing value is the mean of that particular velocity when it is visible, the fit of the heuristic model to the data in the experiment is shown in Figure 4. Filling in allows the model to predict slightly above chance performance in the motor-object occluded condition, but the predictions for the projectile-object occluded condition still miss qualitative aspects of the data especially when *e* = 0.1.

A second possibility, one that abandons the G&P model's collection of heuristics, is that people use a pure logistic regression based on the observable velocities. Logistic regression using a linear combination of the observed velocities must be an approximation because the correct combination rule is nonlinear. This model does very well, predicting all of the main qualitative effects of the data, using an intercept of −1.0, a weight of −0.10 for *u*_*a*_, a weight of 0.85 for *v*_*a*_, and a weight of 0.78 for *v*_*b*_. The signs of the coefficients are in the same direction as in Equation 2, but the magnitudes of the coefficients allow a better fit to the data. In particular the motor-object bias arises from a smaller absolute weight for *u*_*a*_ than the absolute weights for *v*_*a*_ or *v*_*b*_. Of course while the fit to the data is good, as shown in Figure 4, it is very much a descriptive model because there is no easy explanation for why people would have learned these particular coefficients—training with veridical feedback should result in equal coefficients for each of the velocities.

A third possibility is a weak version of the noisy Newton model, in which people directly approximate the noisy Newton model using an approximation from computer science and statistics. For the mass judgment task, an approximation called importance sampling is appropriate. With importance sampling, memories of previous collisions are used as a stand-in for an explicit model of Newtonian mechanics, similar to the way that an exemplar model would (Cohen, 2006; Shi et al., 2010; Sanborn et al., 2013)^3^. For the mass judgment task, the importance sampling approximation works by first comparing the current collision to each remembered to collision to determine the probability that they are the same. Then the judgment is based on the summed probabilities that the current collision is the same as the memories in which the motor object was heavier compared to the summed probabilities that the current collision is the same as the memories in which the projectile object was heavier. Using memories to approximate inference could also explain a deviation from the strong version of the noisy Newton model: people are more accurate when classic intuitive physics tasks are presented in more naturalistic contexts (Kaiser et al., 1985, 1986). This could result from importance sampling if the probability that the current situation is the same as previous experiences depends on surface features of the task. Each simulated participant took 50 noisy samples from the prior (using the same psychophysical noise assumed for the noisy Newton model) and the average over 500 simulated participants matches both the noisy Newton predictions as well as the human data, as shown in Figure 4.

The fits of these three intermediate possibilities illustrate how much the data tell us about the correspondence between people's judgments and physical law: it is strong enough to eliminate the G&P model and very close cousins, but not strong enough to distinguish between algorithmic-level models, such as the logistic regression model or the importance sampling approximation, that have a closer correspondence to the noisy Newton model.

More generally, this work stands as a response to a particular critique about Bayesian models of cognition, namely that Bayesian models provide just-so stories, do not predict new behavior, and are rarely directly compared to extant heuristic models (Sakamoto et al., 2008; Jones and Love, 2011; Bowers and Davis, 2012a,b). Bowers and Davis (2012b) in particular described the need for Bayesian models that make novel predictions that existing non-Bayesian models miss. This work points toward one type of design, namely those with complex interactions between variables and missing information, for which Bayesian models may well predict interesting new behavior better than existing heuristics.

## Author note

The author thanks Tom Griffiths for helpful discussions and Xenia Millar, James Tripp, and Takao Noguchi for collecting pilot data. Adam N. Sanborn was supported by a grant (number ES/K004948/1) from the UK Economic and Social Research Council.

### Conflict of interest statement

The author declares that the research was conducted in the absence of any commercial or financial relationships that could be construed as a potential conflict of interest.

## Footnotes

^1^A version of the G&P model that loosens the assumptions about when heuristics can be used is presented in the discussion.

^2^The projectile object bias does not occur if there is a much smaller standard deviation of the Gaussian prior on velocity or a much larger standard deviation of the noise.

^3^Other proposals are that people have internal physical simulators available for making these judgments (e.g., Hamrick et al., 2011; Smith and Vul, 2013).
