# Supplementary material for: Testing Bayesian and heuristic predictions of mass judgments of colliding objects
Source: Front Psychol. 2014 Aug 26;5:938. doi: 10.3389/fpsyg.2014.00938 (PMC4143616; doi:10.3389/fpsyg.2014.00938)
Supplement: Supplementary file 1 [file DataSheet1.DOCX]

**Appendix**

The Appendix provides additional details about the heuristic and noisy Newton models.

**Model Details**

**Heuristic Model.** The heuristic model makes the response indicated by either a single salient heuristic or uses the default heuristic when both are salient. While the salience function that determines heuristic availability was left undefined by Gilden and Proffitt (1989), I follow Cohen (2006) and generate predictions using salience thresholds. The salience threshold for the ricochet is the change in angle large enough to be noticed. Because the collisions were one dimensional, any reversal was considered salient. For the final speed heuristic, the best fitting parameters from Sanborn et al. (2013) were used: a final speed ratio salience threshold of 1.58, a probability of using the final speed heuristic over the ricochet heuristic of 0.19, and a probability of unbiased guessing (instead of following heuristics) of 0.17.

**Noisy Newton Model.** The noisy Newton model infers the values of states of the world, such as mass, using the noisy sensory data. Using Bayes rule, the probability of the state of the world, *S*, given the observed sensory data, *O,* is $p\left( S | O \right)=\frac{p\left( O | S \right)p(S)}{p(O)}$.

where $p\left( O | S \right)$ is the probability of the observed sensory data, $p(S)$ is the prior probability of states of the world, and$p(O)$ is a normalizing constant.

The prior probabilities on the state of the world are constrained by Newtonian mechanics: the same relationships that hold between the initial and final velocities, the coefficient of restitution, and the masses (assuming point masses and no outside forces) were assumed to hold in the prior. To complete the specification of $p(S)$, the same simple prior distributions used in Sanborn et al. (2013) were placed on all of the variables here. Initial velocities, where positive values reflected rightward motion on the screen and negative values leftward motion, were normally distributed with mean zero and standard deviation of 2, reflecting the belief that slower velocities are more likely than faster velocities (Stocker & Simoncelli, 2006; Weiss et al., 2002). The masses were exponentially distributed, so that lower masses were considered more likely than higher masses. Because the final velocities only depend on the ratio of the masses, the parameter of the exponential distribution has no effect, so the prior does not depend on the units used for mass. The prior for the coefficient of restitution is uniform over its range from zero to one. Finally, the prior distributions on the final velocities are calculated from the prior distributions on the other variables using the equations of Newtonian mechanics.

The noise in each variable, $p\left( O | S \right)$, was psychophysically motivated (Hick, 1950; Notterman & Page, 1957). The observed values were the true underlying Newtonian mechanical values combined with noise that approximately followed Weber’s law: a standard deviation of the noise increasing linearly with the value of the variable. The implementation followed Sanborn et al (2013). First each velocity was transformed into log space: the absolute velocity was multiplied by 0.15 and one was added before the log transformation was applied to the so that the nonlinearity was mild. The sign was then restored. Next a fixed amount of Gaussian noise was added (mean 0 and standard deviation 0.1), and finally the log transformation procedure was reversed.

The state of the world, $p\left( O | S \right)$, inferred in this task was the probability that object $a$ was heavier than object $b$, given the priors and shown velocities. Each response was chosen according to its probability – this probabilistic choice approximates the combination of noisy perception with a deterministic decision rule that always chooses the more likely response.
